# The Next Frontier in the Study of Noncovalent Bonding: Transition Metals

**DOI:** 10.3390/molecules30173643

**Published:** 2025-09-07

**Authors:** Steve Scheiner

**Affiliations:** Department of Chemistry and Biochemistry, Utah State University, Logan, UT 84322-0300, USA; steve.scheiner@usu.edu

**Keywords:** spodium bond, regium bond, matere bond, osme bond, NBO, AIM

## Abstract

As work continues unabated in the study of noncovalent bonding, particularly σ-hole bonds, new challenges have emerged as the participation of transition metals in interactions of this sort is fast becoming appreciated. While there are certain similarities with the halogen, chalcogen, etc, bonds, in which the main group elements participate, there are certain unique properties of these metal atoms that must be analyzed before a complete understanding can be attained. As one example, these atoms tend to act simultaneously as both electron donors and acceptors, a synergistic action that amplifies the overall bond strength. Ideas are expressed in this paper to hopefully guide future work in this exciting new arena.

## 1. Introduction

The hydrogen bond (HB) is perhaps the most prevalent and important noncovalent bond in all of chemistry and biology, engendering periodic books and monographs to summarize the continuing research into its nature and implications [1,2,3,4,5,6,7,8,9,10,11]. So many processes take place in aqueous solution, and one cannot even begin to discuss them without some understanding of the H-bonds that involve water molecules. In addition to these chemical processes, there is the very structure of the molecules, which is partly a product of the H-bonding to the surrounding solvent molecules. From an internal perspective, the geometry adopted within proteins, such as the α-helix and β-sheet, is guided in large measure by the HBs between the peptide groups, as well as certain amino acid sidechains. The storage and transmission of genetic information relies upon the HBs that occur within the nucleic acid base pairs. Its widespread occurrence and overwhelming importance have motivated more than a century of intense scrutiny of the HB, which has led to a solid understanding of the roots of its stability, geometrical preferences, factors that affect its strength, and spectroscopic manifestations.

Although hints of their existence emerged in the literature some years ago, it is only in the last two decades or so that the existence of a set of noncovalent bonds, very closely related to the HB, has been appreciated. These bonds differ from the HB by the replacement of the bridging proton by another element, typically drawn from the right side of the periodic table. As its name implies, the halogen bond (XB) substitutes H with a halogen X atom: Cl, Br, or I. This sort of bond was initially deemed counterintuitive, as the electronegativity of X would impart to this atom a partial negative charge, in contrast to the positive H. As such, it was difficult to envision how it might attract a nucleophile, with its partial negative charge. This apparent paradox was resolved upon closer scrutiny of the electrostatic potential surrounding X. While it does indeed contain an overall negative charge, this equator of negative charge, which encompasses the X lone pairs, surrounds a positive region along the extension of the R-X bond axis. Formation of the RX σ-bond tends to deplete the electron density along the bond extension. As the electrons carry a negative charge, a reduced electron density cannot adequately compensate for the highly positive potentials emanating from the nuclei, so it translates to a positively charged region, which was christened with the sobriquet of σ-hole, a name that has stuck.

The halogen bond is now understood to represent only one of a larger set of noncovalent bonds, collectively known as σ-hole bonds [12,13,14,15,16,17,18,19,20]. Subsequent work has demonstrated that the XB is not unique in its formulation, in that X can be replaced by other atoms of varying electronegativity. Depending upon the group of elements from which this replacement is drawn, these other σ-hole bonds are generally denoted under the heading of chalcogen, pnicogen, tetrel, and triel bonds [21]. Like the hydrogen [22] and halogen bonds [23], some of these closely related cousins have been formally recognized and defined by IUPAC [24,25]. Their differences from the XB are minor, the chief being the number of σ-holes associated with each central atom. For example, the divalent structure of many YR_2_ molecules endows the chalcogen with two separate σ-holes, each lying roughly opposite a RY bond. One feature of these bonds is that they usually do not include first-row atoms, viz. F, N, O, C. The high electronegativity of these atoms, coupled with their low polarizability, makes them resistant to the formation of a σ-hole.

As work in this area progressed, it was soon realized that a further extension of these concepts was warranted. It is not uncommon for one of these atoms to occur within the framework of a planar molecule. One obvious example arises in the context of the formaldehyde analogues R_2_TO, where T represents a tetrel atom. A positive region of the electrostatic potential is found above (and below) the plane of the molecule, located roughly above the T center. In analogy to the σ-hole idea, these positive regions of density depletion are generally referred to as π-holes [26,27,28]. Such positive regions do not necessarily associate themselves with a single atom. An example that comes immediately to mind is the π-hole that develops above the center of the hexafluorobenzene molecule, equidistant from all six C nuclei.

The large and rapidly accumulating body of work dealing with these σ- and π-hole bonds has led to a great deal of insight into the fundamentals of their bonding. For example, the Coulombic interaction between the two molecules represents a substantial component of their stability but does not stand alone. Another contributor is derived from a certain amount of charge transfer from the approaching nucleophile (usually a lone pair) to the antibonding σ*(RA) orbital, where A represents any of the halogen, etc, atoms that have replaced H. The accrued density in this antibonding orbital typically leads to a RA bond weakening and lengthening, with secondary consequences for the vibrational spectrum. Bond strength usually leads back to the depth of the σ-hole, which in turn is amplified by the presence of electron-withdrawing substituents on the Lewis acid electrophile. Another means of manipulating bond strength derives from cooperativity. If the lone pairs of the X atom are being used as electron donors in an HB, for example, the loss of total density on X is reflected in a more positive X σ-hole, which can, in turn, make this X a more potent electron acceptor within an XB [29,30,31,32].

## 2. Participation of Transition Metals

A more recent turn of events occurred when it was gradually realized that the list of elements that could participate as HB proton substitutes extended to the d-block transition metals [33,34,35,36,37,38,39,40,41,42,43,44,45,46,47,48,49,50,51,52,53,54]. In hindsight, this makes perfect sense and could probably have been anticipated. These atoms are electropositive metals, so it would not be a stretch to imagine positive regions nearby, whether σ- or π-hole in character. They are also rather polarizable, which would tend to add to their ability to generate such holes. Another feature of these atoms is the number of formal oxidation states that characterize them. Also, the shape of their coordination shell is quite variable. For example, the transition metal M atom in a tetravalent MR_4_ bonding situation might be tetrahedral, planar, or some other shape.

**Figure 1:**
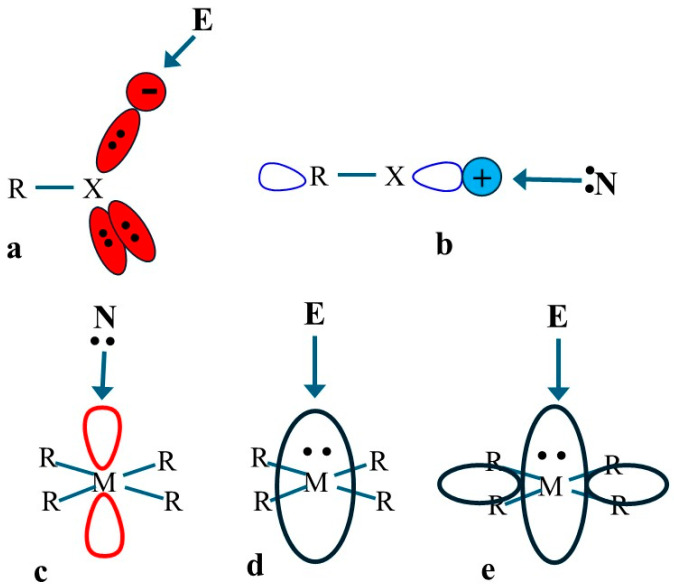
Cartoon depiction of disposition of lone pairs, molecular electrostatic potential extrema, and interacting orbitals.

In the case of the main-group elements, their electronegativity allows them to participate in noncovalent bonds in the capacity of either an electron donor or acceptor. Taking a halogen atom X within a single-bonded R-X scenario as an example, this atom is characterized by three lone pairs, designated by the red areas in Figure 1a, each containing two electrons. An electrophile E can approach any of these pairs, which would place its optimum approach at perhaps some 60° from the R-X axis extension. This interaction is aided by the negative charge around the lone pair, indicated by the red circle, which will attract the electrophile.

As another aspect of the electronic structure of RX, there is a vacant σ*(RX) antibonding orbital, indicated by the empty shapes on either side of the molecule in Figure 1b. A nucleophile N can approach this orbital along the R-X extension so as to donate some of its density to this orbital. This approach is also guided by a positive region in the RX potential, the blue + symbol in Figure 1b, which helps to draw in N. It is important to stress at this point that one can easily guess whether an RX is acting as an electron donor or acceptor, based purely on the geometry of the complex, i.e., the angle of approach of the second molecule.

The situation can become more complicated for transition metals. The square planar arrangement around M within the MR_4_ system serves as an example, where M represents any of the group 10 metals Ni, Pd, or Pt. The central metal M contains several orbitals of particular interest. Within the natural bond orbital (NBO) localization scheme, there is an empty orbital that resembles p_z_, as depicted in the cartoon of Figure 1c. A nucleophile N would be attracted to this orbital, so it would prefer to approach along the perpendicular *z*-axis. As a second factor, the M also contains two filled orbitals, illustrated in Figure 1d,e, very roughly, approximating d-orbitals in shape. These orbitals can donate density to an approaching electrophile, which would again prefer the same *z*-axis for its position. Clearly, then, a ligand would tend toward the same *z*-axis position, whether acting as an electrophile or nucleophile. So, the geometry of the complex would offer little in the way of guidance as to which subunit is a donor and which is an acceptor.

One might suggest the distinction might be made based on the character of the approaching ligand. A nucleophile will be drawn in only insofar as it might donate density, so the metal complex must be acting as an electron acceptor. In parallel logic, an electrophilic ligand must be drawing density from the M, which is thus an electron donor. While this logic is appealing, it ignores the fact that many ligands may not be solely nucleophilic or electrophilic. Consider the FI molecule as a prime example. The electron-withdrawing F induces a σ-hole on I, which readily acts as an electron acceptor through the σ*(FI) orbital. But at the same time, the I lone pairs are primed to donate charge. The FI molecule is hardly unique in this regard. There are numerous examples of molecules where a source of electron density, be it a lone pair or π-region, is a close neighbor of a positively charged region or an antibonding orbital.

Another idea that is worth considering is the sign of the potential in the region of interest. It might be thought that if a positive region serves as an unambiguous signal, then this segment of the molecule must act as an electron acceptor, and vice versa if the potential is negative. While this concept is attractive, it is not borne out of observation. The planar square MR_4_ systems, where M is a group 10 metal, argue against this purported rule. Calculations show that the potential of the area directly above M varies from +13 to 0 to −8 kcal/mol when M is Ni, Pd, and Pt, respectively [55]. Yet the interaction energy of each molecule with ICCH, with its I lying directly above M, is barely affected by this change in the sign of the potential. More importantly, there is a net transfer of charge to the metal system from ICCH in all cases, even when the charge above M is negative, and one might have expected the M to act as nucleophile.

One can thus see an important complication arising in transition metals. In the main-group situation, one can usually distinguish whether the X serves as an electron donor or acceptor by the approach direction of the partner molecule. The σ*(RX) antibonding orbital lies directly along the RX axis, so a nearly linear RX··N alignment is indicative of X acting as an electron acceptor, the definition of a halogen bond. The lone pairs are situated roughly 60–70° from this axis, so a substantial deviation in the partner molecule toward a lone pair is highly suggestive that X is an electron donor, i.e., not an XB. So, the geometry is highly predictive. Considering the group 10 metals described above, on the other hand, the M lone pair is oriented along an axis perpendicular to the molecular plane. But the vacant p_z_ orbital occupies this same space. So, the positioning of another molecule directly above M results in an ambiguous situation. From the geometry alone, one cannot tell which of these orbitals is participating to a larger extent, and whether M is primarily a donor or acceptor.

## 3. Resolving Direction of Electron Transfer

The important question arises as to how to tell whether the metal atom is acting as an electron donor or acceptor. And if serving in a dual capacity, what means of analysis can discern which is primary? While the Quantum Theory of Atoms in Molecules (QTAIM) [56] has found real use in identifying the presence of bonding interactions between molecules, it is less straightforward in terms of determining the direction of charge flow. So again, taking the M··I interactions described above, the QTAIM certainly finds there to be a bond of some sort here, but is unclear as to whether it would be best described as semicoordinate (M acts as an electron acceptor) or halogen. This same limitation applies to pictorial representations of the reduced density gradient, or noncovalent interaction (NCI) analysis, which indicates noncovalent bonding through a three-dimensional color scheme [57,58,59], but makes no distinction as to direction of charge flow.

As one fairly straightforward means to make this decision, one can simply consider the total of the atomic charges on each subunit. A negative total on subunit A would indicate a net transfer of density from B to A. There are, of course, a multitude of formulations as to atomic charge, none of them considered to be definitive. But one can consider this charge sum within several of these frameworks, to ensure all provide the same overall direction of density shift, even if they differ quantitatively.

The second scheme that is useful in this regard is the natural bond orbital (NBO) scheme [60,61]. This procedure localizes the total electron density into individual orbitals that typically correspond to σ and π bonds and lone pairs, as well as virtual orbitals with small fractional occupations. For example, the halogen bond is commonly characterized within this framework by charge transfer from a lone pair of the nucleophile into the σ*(XR) antibonding orbital of the Lewis acid. The presence of such charge transfers, as well as the magnitude of E2, the second-order perturbation energy associated with this transfer, is a preeminent marker of halogen bonding. On the other hand, when the approaching ligand donates density, this charge transfer will manifest itself via combinations of occupied orbitals on the ligand with vacant orbitals on the M system. A problem that may arise in the application of this approach occurs when the bonding becomes stronger. When that is the case, the NBO scheme fails to separate the full complex into two separate subunits, clouding its interpretation.

Another idea could be the pictorial depiction of density flow by the generation of electron density difference maps. These maps display the difference between the total density of the complex and the sum of the densities of the two subunits. However, in addition to the charge transfer from one subunit to the other that could help characterize the bond type, these maps also contain the internal polarizations within each monomer. The latter tend to be of larger magnitude than the former, complicating the interpretation of such density difference diagrams.

## 4. Examples

**Figure 2:**
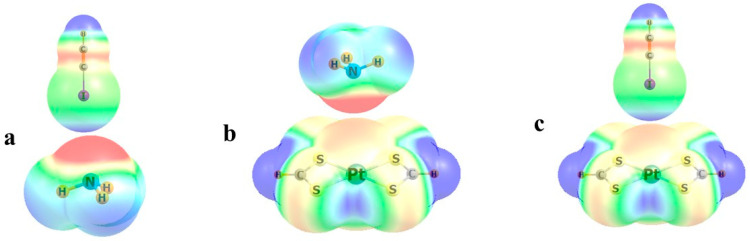
Molecular electrostatic potentials of monomers within complexes (**a**) H_3_N···ICCH, (**b**) H_3_N···Pt(SCHS)_2_, and (**c**) HCCI···Pt(SCHS)_2_. Red and blue regions, respectively, indicate negative and positive potentials.

**Figure 3:**
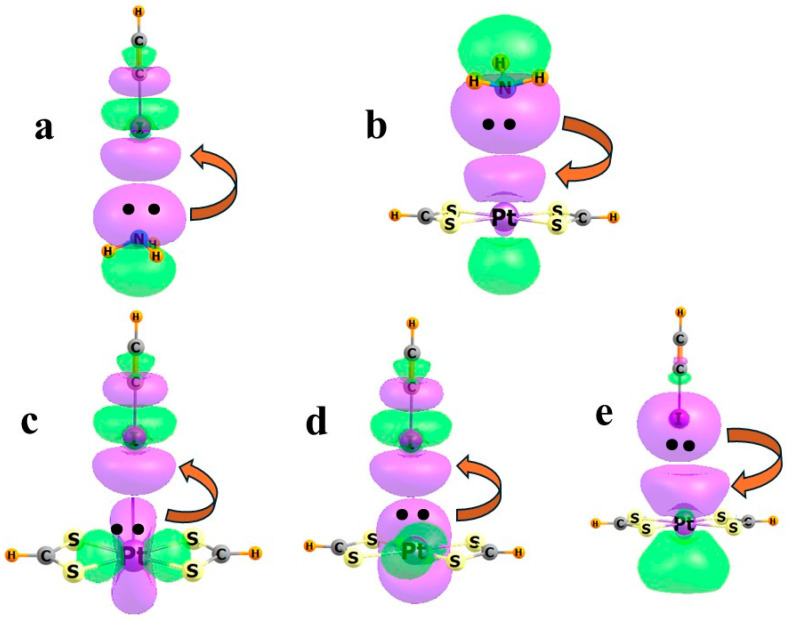
Interacting localized orbitals in (**a**) H_3_N···ICCH, (**b**) H_3_N···Pt(SCHS)_2_, and (**c**–**e**) HCCI···Pt(SCHS)_2_. Electrons are shown as dots, and direction of charge transfer is shown by red arrows.

These points can be made more concrete by way of a few simple examples. Figure 2a displays the molecular electrostatic potential (MEP) of the two subunits when ICCH is combined with NH_3_ to form a HCCI··NH_3_ halogen bond. These computations and those below were conducted with the M06-2X DFT functional in the context of the def2-TZVP basis set. The negative red area above N is coincident with its lone pair, which is transferring charge to ICCH. The blue region below I indicates its positive σ-hole, coincident with its σ*(IC) antibonding orbital as the charge recipient. This transfer is characterized in Figure 3a.

An example of the issue as it pertains to transition metals arises when the square planar Pt(SCHS)_2_ system is combined with NH_3_, as in Figure 2b. There is a slight negative charge directly above the Pt, which tends to repel the negative region near N, but this mild repulsion is overcome by other factors. In particular, there is a substantial overlap between the N lone pair and the vacant p_z_ orbital on Pt, which results in the charge transfer indicated by the curved red arrow in Figure 3b. The NBO scheme evaluates the second-order perturbation energy E2, associated with this transfer, to be 15.1 kcal/mol. Notably, this is the only intermolecular transfer of any substance, so there is no ambiguity associated with the question as to which molecule acts as the donor or acceptor. The overall direction of charge transfer is confirmed by the total of atomic charges on each subunit, amounting to 0.045 e, with NH_3_ assuming an overall positive charge. It may be noted that it is the p-orbital of Pt that is the recipient of the charge transfer, rather than d_x_2−_y_2, which might be expected to be lower in energy. This observation may be due to the superior overlap between the N lone pair and the Pt p_z_ orbital.

The story becomes a bit more complicated when ICCH is placed in coincidence with Pt(SCHS)_2_, taking on the T-shaped complex shown in Figure 2c [55]. The negative region above Pt coincides nicely with the positive σ-hole on I. There is substantial overlap between the occupied Pt lone pairs shown in Figs 3c and 3d, and the σ*(IC) antibonding orbital. The E2 values for these two transfers sum to 3.4 kcal/mol, all indicating the presence of a halogen bond. However, there is another, and very substantial, component to the binding. Figure 3e documents a strong overlap between one of the I lone pairs and the unoccupied p_z_ orbital of Pt, which is very analogous to the case depicted in Figure 3b, where it is a N lone pair contributing charge. E2 for this transfer overshadows that for the two transfers into σ*(IC), amounting to 21.5 kcal/mol. Consistent with these comparative values, there is a net transfer of 0.028 e from ICCH to Pt(SCHS)_2_.

All this is not to say that there are no elements of halogen bonding present at all. There are indeed appropriate interactions between the M lone pair and the σ*(IC) orbital, with some charge shift in that direction. It is simply that the semicoordinate bonding in Figure 3e makes a larger contribution. One might designate one of the two interactions as back-bonding, since charge moves in opposite directions. As an added bonus, since the two phenomena shift charge in opposite directions, there would be a certain degree of synergistic positive cooperativity that would act to strengthen the interaction overall, not unlike the cooperativity in H-bonding, where a central molecule acts simultaneously as both the electron donor and acceptor.

**Figure 4:**
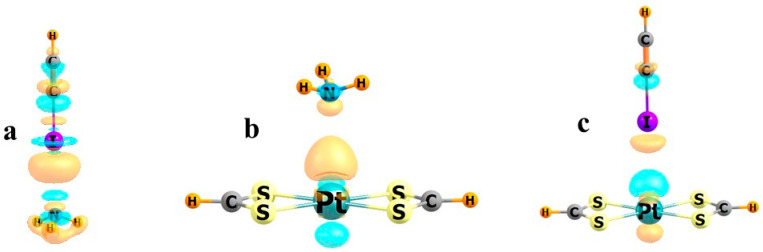
Electron density shifts in (**a**) H_3_N···ICCH, (**b**) H_3_N···Pt(SCHS)_2_, and (**c**) HCCI···Pt(SCHS)_2_. Blue and orange colors represent gains and losses in density, respectively.

With regard to three-dimensional representations of charge flow, the electron density difference maps of three systems are displayed in Figure 4. The HCCI··NH_3_ system in Figure 4a is illustrative of a classical XB. There is an orange region of density loss directly below I, with a smaller blue area of charge accumulation right above N. These features are a result of the polarizations within each subunit, with charge flowing from H to N within NH_3_ to prepare the molecule to interact with HCCI, concomitant with a polarization within HCCI, with density shifting upward internally. Although NH_3_ again serves as an electron donor when paired with Pt(SCHS)_2_, the shift pattern is different in Figure 4b, with an orange region of density loss in its lone pair region. There is also a density depletion above the Pt, as it shifts downward, so as to impart to the region above Pt a more positive potential. But the differences in some of the fundamental features of Figs 4a and 4b, despite the unambiguous activity of NH_3_ as an electron donor, could make it difficult to use such density difference maps as the arbiter of the nature of the bond. As another point, the map for HCCI··Pt(SCHS)_2_ in Figure 4c is reminiscent of Figure 4a, so it might suggest the presence of an XB. However, while there are some indicators of halogen bonding, this complex is held together primarily by a semicoordinate bond, so this similarity might be misleading.

This dual charge transfer is not limited to the particular XCCH··Pt(SCHS)_2_ system mentioned above. Replacement of the HCCX with FX yields a similar result. There is a charge transfer into the σ*(XF) antibond, which is overshadowed by a larger E2 for transfer from the X lone pairs to Pt. Moreover, the findings are not limited to the approach of a halogen atom on the partner molecule. A recent set of computations [62] extended these ideas to groups 14–16.

From an experimental perspective, an earlier study [63] observed that I atoms approach a Ni within a square planar arrangement closer than their van der Waals (vdW) radii sum, and the accompanying survey of the Cambridge Structural Database (CSD) yielded 50 crystal structures of this sort, where Ni lies close to a halogen or O atom. In a very recent report [64], Pt was placed in a somewhat different square planar arrangement with I as two of its four surrounding ligands. This Pt was able to interact attractively with the I of I_2_ or some other I-containing species. However, the approaching I atom was displaced from a position directly above the Pt, sliding along the Pt-I covalent bond toward another I center. A persuasive confirmation of the ideas emanating from the calculations discussed here comes from a recent report by Eliseeva et al. [65], who concluded from crystal structures coupled with quantum calculations that the bonding of the Pt in a square planar arrangement could be either of two sorts. Its d_z_2 orbital could donate charge to I in the context of a classic halogen bond. But with a change in intermolecular alignment, the Pt p_z_ orbital could accept charge from chalcogen S lone pairs to constitute a semicoordinate bond. The dominance of the semicoordinate bonding cannot be taken for granted as a universal theme. Lin and Gabbaï, for example, constructed systems [66] that contained an internal noncovalent Pd··Te where the dominant theme was shown to be chalcogen bonding, wherein the square planar Pd center acts as an electron donor. Another study [67] obtained a basically similar result, in that a Pt··Te chalcogen bond characterized by transfer from a Pt d_z_2 orbital was somewhat more important than the semicoordinate bond where charge was transferred from the Te lone pairs to Pt. One can thus conclude that both sorts of bonding are generally present, and that the precise balance will depend on the particulars of the systems and their geometries.

A different modification retains the planar configuration of the Pt, but replaces four Pt-S attachments arising from the two ditopic SCHS ligands with a ditopic modified phenanthrene and a pair of Cl atoms [68]. When combined with a BrF unit, there is an NBO E2 of 27.5 kcal/mol for transfer into the σ*(BrF) from the Pt lone pair orbital, symptomatic of a halogen bond. However, there is also a net E2 of 65.0 kcal/mol for transfer in the opposite direction, from the Br lone pair into vacant orbitals on Pt. Despite the larger size of the latter, there is an overall transfer of 0.094 e from the Pt system to BrF. This is a case where the overall charge transfer conflicts with the relative magnitudes of E2, so there is some ambiguity as to the dominant bonding type. But importantly, it confirms the dual nature of the binding, with some charge flowing in both directions. These ideas concerning the dual nature of the bonding find support in the recent literature, arising from the analysis of a number of crystal structures and their theoretical analysis [63,65,69,70,71,72,73,74,75].

This situation applies to anionic systems, as well. A crystal structure pairs tetrachloridopalladate(II) (PdCl_4_)^2-^ dianions with each other in such a way that a Pd-Cl bond lies directly above the Pd center of another dianion, pointing down in such a way that it would appear to signify a Pd-Cl··Pd halogen bond [76]. However, calculations show that there is a net transfer of charge of 0.032 e to the lower of the two anions, from the Cl lone pair to the lower Pd center, which serves as an electron acceptor. E2 for this transfer is equal to 12.5 kcal/mol, as compared to 3.3 kcal/mol for the halogen bond-associated transfer to σ*(Pd-Cl). Another work [77] confirmed the tendency for the M center to serve as an electron acceptor from a halogen lone pair, this time in the context of CuCl_4_^2–^ and CuBr_4_^2–^.

## 5. Covalent or Noncovalent

Most of the main-group noncovalent bonds are clearly much weaker than their covalent counterparts. The interatomic separations are much larger than the sum of the covalent radii of the two interacting atoms, and only a bit smaller than the sum of their vdW radii. This distinction becomes a bit fuzzier for transition metals. Given the variability in the number of surrounding ligands for a given metal atom, and the shape adopted for the system, as well as the different spin multiplicities that are possible, the assignment of a covalent or van der Waals radius to each atom is rather nettlesome.

The criterion of interatomic distances is prone to another, more fundamental complicating issue in the context of transition metals. The M-L distances to ligands tend to be on the same order as those involving the presumed noncovalent bond. The data in Table 1 expands on this issue. A series of electrically neutral ML_n_ chlorides was allowed to interact with NH_3_ [78], and the binding energies of each are listed in the table. The next two columns contrast the internal M-Cl bond length with the intermolecular M··N contact distance. The similarities are striking, with only small differences between the two. If each M-Cl contact is considered covalent, then one would be tempted to make the same assignment for M··N, even though these interaction energies are not all very large; many are less than 30 kcal/mol. Of course, N is smaller than Cl, so the similar distances speak to a slightly weaker M-N bond. To be a bit more quantitative, the vdW radius of N is less than that of Cl by 0.16 Å [79], so one might expect the M··N distances to be shorter than M··Cl by this same amount, given the equally strong bonds.

While AIM does not directly monitor the direction of charge transfer, it does have the potential to assess the strength of a given bond, whether covalent or noncovalent. In particular, the value of the density ρ_BCP_ at a bond critical point allows for comparisons between bonds and has even been applied within the context of simple relationships to provide an energetic estimate of each [80,81,82,83]. In a similar vein, the total energy density H_BCP_ at that point can serve a similar purpose. In fact, the sign of H_BCP_ is commonly taken as a test of covalency, i.e., a negative H_BCP_ is an indicator of covalency [84,85,86]. These two indicators are displayed in Table 2 for the same complexes with NH_3_ as in Table 1. With respect first to ρ_BCP_, its values for the M-Cl bonds are generally around 0.1 au with a few exceptions. These same quantities are generally smaller for M··N, but fairly large nonetheless, greater than 0.06 au. The exception occurs for RuCl_2_, where the two values of ρ are nearly identical. One would probably conclude that the M··N bonds are only slightly weaker than the coordinate M-Cl. If one were to accept an arbitrary cutoff for covalency to be 0.04 [87], most of these bonds would be classified as covalent. An opposite conclusion might be derived if the delimiter were set at 0.1 [88]. The possible covalency of these M··N bonds is reinforced by the universally negative values of H_BCP_, generally in the range between −0.01 and −0.02 au.

Given the arbitrariness of these thresholds, as well as the lack of clear defining characteristics, it is perhaps best to abandon the idea of a sharp distinction separating covalency from noncovalency. Rather, this concept might be best imagined as a spectrum, where these two categories smoothly segue from one to the other. But importantly, there is a tendency for the interactions involving transition metals to be rather strong, containing high notes of covalency, comparable to the bonding to the other ligands. It might be worth pointing out that the systems listed in Table 1 and Table 2 might be deemed “coordinatively unsaturated” since they contain vacant sites available for further coordination to a ligand. This situation differs in some respects from the tetracoordinated group 10 square planar systems described above.

## 6. Spin Multiplicity

Unlike most situations involving main-group elements, which are part of singlet states, transition metals frequently occur in states of higher multiplicity. Further, the energy separations between different spin states can be fairly small. So, it would be good practice in calculations of such systems to check whether the suspected multiplicity is indeed the most stable. A number of examples were provided in a recent set of calculations [78] involving MCl_n_. The triplet state of MoCl_2_ is more stable than the singlet by 23 kcal/mol, and the quintuplet is lower in energy by 46 kcal/mol. With regard to the interaction energies with NH_3_ as a nucleophile, the interaction energies are nearly independent of the multiplicity. These quantities are equal to 37.5, 35.1, and 36.8 kcal/mol for the singlet, triplet, and quintuplet, respectively. This near uniformity, however, is not a universal rule. Using TcCl_3_ as a counterexample, the triplet and quintuplet lie lower in energy than the singlet by 31 and 57 kcal/mol. The interaction energies of these three states with NH_3_ are 55.6, 31.3, and 55.8 kcal/mol. So, for this system, the triplet binds considerably more weakly than do the singlet or quintuplet. As an example of where the two states of the monomer are close in energy, the triplet state of PdCl_2_ lies only 4 kcal/mol lower in energy than its singlet. Yet there is a bit larger difference in interaction energies, 37.7 kcal/mol for the singlet and 30.6 kcal/mol for the triplet.

## 7. Nomenclature

As with many new concepts, one is frequently faced with the question of what to call them, an issue that has been wrestled with in recent years [21,89,90,91]. It is preferable for the labels affixed to them to provide some basic information as to the source of their strength and the atoms involved. The noncovalent bond where H serves as a bridge atom led to the title of hydrogen bond, for which the contributing factors are now well understood to involve both coulombic and charge transfer effects. The substitution of H by Cl, Br, or I led to the introduction of halogen bonding as a natural naming extension. As this sort of bonding bled over to the other main group elements, this idea was further propagated by the titles of chalcogen, pnictogen, tetrel, and triel bonds. Of course, the common characteristics of these interactions have led to proposals that they all be lumped into the overarching category of σ-hole bonds.

With regard to the d-block elements, this philosophy of providing a different name for each family has been continued to some extent. Names such as spodium [33,34,35,36,37,92,93,94,95], regium [38,39,40,41,42,43,96,97], and wolfium [50,98] bonds have been proposed for various of these bond types. This variety of names has the advantage of immediately identifying the atom type that is directly involved. An alternate philosophy might adopt the idea applied to σ-hole bonds of the p-block: acknowledge their similarities by lumping them under the blanket heading of semicoordinate bonds, as proposed earlier [63,67,69,70,99]. This name extends the idea of coordination of transition metals, but also conveys that these bonds tend to be weaker than standard coordinate bonds.

Regardless of whether applying the specific appellation of spodium, regium, etc, or the more general semicoordinate designation, or even the main group names, such as σ-hole, halogen, and so on, all of these names presuppose that the atom in question is serving as an electron acceptor. But any of these labels would be incorrect and misleading if the metal is an electron donor, in which case the interaction would be classified entirely differently, according to the atom of the ligand that accepts the charge from M. As mentioned above, the finding of an attractive interaction between M and another atom via the AIM, NCI, or some variant of these does not supply the requisite information as to which atom acts as an electron acceptor. So, a more detailed examination of an M··I bonding interaction, for example, would be required before designation as either semicoordinate or halogen bonding.

Why does it matter which label is used, since the bonding is present regardless of its title? There is, of course, the natural desire for a complete understanding of the fundamentals of the attraction. But more than that, there are practical reasons, as well. The type of interaction affects how one might engineer the system to amplify or attenuate the bond. Were M to serve as an electron acceptor, adding electron-withdrawing substituents to M would enhance both its σ or π-hole and its ability to store additional density that would be transferred to it from an approaching nucleophile. Conversely, it would be electron-donating substituents that would be required to strengthen the bond if M were to act as an electron donor. Taking the M(SCHS)_2_ systems above as an example, if theoretical analysis were to find that M is acting as an electron donor, the interaction would likely be strengthened if the ligands were more electron-donating. The H atom in the ligands might perhaps be replaced by alkyl or OR groups. Conversely, the interaction would be strengthened by electron-withdrawing substituents like CN or NO_2_ if M is serving as an electron acceptor.

An example of mislabeling and how it might lead to misunderstanding of an interaction arises in the recent discussions concerning the stabilizing interaction between a SiH group and a halogen atom in another molecule. The differing electronegativity between Si and H leads to Si^+^-H^-^ polarization, so it was proposed [100,101] that this interaction constitutes a hydridic SiH··X H-bond with the halogen, an unusual and unexpected phenomenon. Such a bond would imply charge transfer to SiH from X. However, more detailed scrutiny [102,103,104,105,106] later demonstrated charge flow in the opposite direction, viz., from the σ(SiH) bonding orbital to the σ*(XR) antibond, an unmistakable sign of a halogen bond. This conclusion was amplified by the molecular geometries, which showed a near-linear RX··H in combination with a distinctly nonlinear SiH··X alignment.

Of course, the examples described above, and those from several other researchers, show that the situation is not always so simple. There are examples where M serves as both an electron donor and acceptor simultaneously. These situations complicate the choice of name for the stabilizing interaction. One might choose the label based on the overall direction of charge transfer, i.e., the more prominent of the two. Or perhaps, the interaction could more accurately be typed by a hybrid designation such as semicoordinate/halogen. But regardless of how one chooses to refer to the bond in question, it is important to convey to the scientific audience that the interaction goes beyond a simple name, whose complexity must be understood.

Another question that arises concerns the borderline between a covalent and noncovalent bond. A number of researchers have wrestled with this question and proposed certain thresholds [87,88,107,108]. One criterion concerns the energy of interaction. While a value of 5–10 kcal/mol would certainly suggest noncovalency, and a value above 80 kcal/mol would clearly point toward a covalent bond, it is the intermediate set that is more problematic. Perhaps the 30–50 kcal/mol range might be suggested as a border that smoothly transitions between these two designations. Another marker might arise in the context of the QTAIM. Bond critical point densities that are larger than 0.04 to 0.06 might serve as an alternate boundary.

The examples presented here have focused attention on square planar arrangements with the transition metal in its center. But this, of course, represents only one of many geometries that this family of metals can adopt. Mo, for example, can occur within a near-linear molecule in the context of MoCl_2_, or a tetrahedral arrangement in MoCl_4_ [78]. The former would permit the approach of a ligand from a direction above the Mo, while this ligand would have an opening to approach a face of the tetrahedron in the latter. A similar line of approach would apply to the linear HgR_2_ and ZnR_2_ systems [95]. The PdCl_2_ molecule is highly bent, so a ligand might best approach opposite one of the Cl atoms. PdCl_4_, on the other hand, adopts a distorted octahedral geometry upon binding a fifth ligand. CdCl_4_ is different in that its structure is nearly square planar, so a ligand adopts the apical position in a square pyramid. TcCl_3_ exists as a planar triangle, so a ligand might approach from directly above the Tc, comparable to the planar systems discussed above, and a similar sort of motif would arise in connection with NbCl_3_ [78]. Several other planar motifs were explored for the regium atoms Cu, Ag, and Au, and how ligands might best approach them [109]. MoF_4_O occurs in a square pyramid shape, from which a ligand might approach from directly below the O at its apex [50]. The tetrahedral shape of OsO_4_ leads to an opening at the center of each tetrahedral face [45].

## 8. Guidance for the Future

The study of the interactions involving transition metals is quickly picking up steam. This work is, in some ways, more complex and subtle than what has been learned about main group chemistry, and for this reason, more rewarding. It will be important to understand that these interactions are prone to invoke multiple sorts of bonding, with charge transferring in two different directions. The metal atom can thus act as both an electron donor and acceptor within the same complex. The geometry of the complex can be misleading, suggesting one sort of bonding, while it is another that is dominant. So, calculations must include means of looking at these two modes separately, perhaps via the NBO scheme or some similar protocol. As a corollary, two subunits can engage in a stable complex, even when the conflicting signs of their electrostatic potential extrema or atomic charges indicate otherwise. The spin state must be seriously considered, as there may be small energetic separations between the dominant and other multiplicities. One can expect many of these so-called noncovalent bonds to be rather strong, comparable to the internal metal-to-ligand bond strengths within the ML_n_ system itself.

With these caveats in mind, researchers are encouraged to accelerate their inquiries into the bonding of transition metals. In many ways, this group of elements represents the new frontier in our understanding of molecular interactions. The implications for inorganic chemistry and crystal engineering are obvious. It will be fascinating to watch the new insights that emerge from this research and the manner in which they will touch other aspects of chemistry.

## Figures and Tables

**Table 1:** Binding energies (E_b_, kcal/mol) and M-Cl and M-N interatomic distances R_Cl_ and R_N_ (Å) within MCl_n_··NH_3_ complexes, calculated at M06-2X/def-pvtz level [78]. All complexes are neutral and in singlet state.

Group	M	n	−E_b_	R_Cl_	R_N_
3	Y	1	25.55	2.440	2.455
		3	39.01	2.475	2.428
5	Nb	1	42.98	2.283	2.261
		3	27.80	2.294	2.302
		5	29.38	2.321	2.356
6	Mo	2	37.28	2.279	2.174
		4	30.20	2.262	2.301
7	Tc	1	41.91	2.234	2.196
		3	54.40	2.241	2.086
		5	23.22	2.262	2.283
8	Ru	2	54.16	2.246	2.054
		4	27.53	2.207	2.206
10	Pd	2	38.03	2.237	2.145
		4	40.15	2.255	2.132
11	Ag	1	30.06	2.328	2.246
		3	40.01	2.292	2.147
12	Cd	2	20.92	2.351	2.412
		4	22.75	2.390	2.422

**Table 2:** Density ρ_BCP_ and total energy density H_BCP_ (au) of MCl_n_˖˖NH_3_ complexes at their bond critical points, calculated at M06-2X/def2-pvtz level [78]. All complexes are neutral and in singlet state.

Group	A	n	ρ_BCP_		H_BCP_	
			M-Cl	M··N	M-Cl	M··N
3	Y	1	0.0734	0.0500	−0.0171	−0.0055
		3	0.0652	0.0503	−0.0120	−0.0047
5	Nb	1	0.1066	0.0781	−0.0407	−0.0182
		3	0.1016	0.0669	−0.0377	−0.0126
		5	0.1009	0.0634	−0.0378	−0.0121
6	Mo	2	0.1008	0.0833	−0.0394	−0.0174
		4	0.1064	0.0638	−0.0403	−0.0095
7	Tc	1	0.1181	0.0781	−0.0461	−0.0155
		3	0.1066	0.0978	−0.0370	−0.0215
		5	0.1109	0.0721	−0.0415	−0.0137
8	Ru	2	0.1035	0.1041	−0.0335	−0.0245
		4	0.1192	0.0743	−0.0454	−0.0113
10	Pd	2	0.1036	0.0801	−0.0315	−0.0122
		4	0.1029	0.0881	−0.0317	−0.0165
11	Ag	1	0.0807	0.0652	−0.0181	−0.0078
		3	0.0912	0.0840	−0.0240	−0.0142
12	Cd	2	0.0770	0.0466	−0.0170	−0.0045
		4	0.0700	0.0457	−0.0146	−0.0044

## Data Availability

Data are available by author upon request.

## Abbreviations

QTAIM	Quantum Theory of Atoms in Molecules analyzes topology of electron density [56].
AIM	Atoms in Molecules: shortened version of the preceding.
NCI	Noncovalent Interactions: pictoral description of reduced density gradient [57].
NBO	Natural Bond Orbital: means of localization of electron density accenting orbitals involved in charge transfer [110].
vdW radius	van der Waals, referring to approximate atom size [79,111,112].
